# Biogeography of Fungal Communities Associated with *Pinus sylvestris* L. and *Picea abies* (L.) H. Karst. along the Latitudinal Gradient in Europe

**DOI:** 10.3390/jof9080829

**Published:** 2023-08-06

**Authors:** Valeriia Mishcherikova, Jūratė Lynikienė, Adas Marčiulynas, Artūras Gedminas, Oleh Prylutskyi, Diana Marčiulynienė, Audrius Menkis

**Affiliations:** 1 Institute of Forestry, Lithuanian Research Centre for Agriculture and Forestry, Liepų Str. 1, Girionys, 53101 Kaunas, Lithuania; valeriia.mishcherikova@lammc.lt (V.M.); jurate.lynikiene@lammc.lt (J.L.); adas.marciulynas@lammc.lt (A.M.); arturas.gedminas@lammc.lt (A.G.); 2 Department of Mycology and Plant Resistance, V.N. Karazin Kharkiv National University, Svobody Sq., 61022 Kharkiv, Ukraine; prylutskyi@karazin.ua; 3 Department of Forest Mycology and Plant Pathology, Uppsala BioCenter, Swedish University of Agricultural Sciences, 75007 Uppsala, Sweden; audrius.menkis@slu.se

**Keywords:** abiotic stress, climate change, forest damage, fungal communities, pathogens, tree species, Scots pine, Norway spruce

## Abstract

We assessed the diversity and composition of fungal communities in different functional tissues and the rhizosphere soil of *Pinus sylvestris* and *Picea abies* stands along the latitudinal gradient of these tree species distributions in Europe to model possible changes in fungal communities imposed by climate change. For each tree species, living needles, shoots, roots, and the rhizosphere soil were sampled and subjected to high-throughput sequencing. Results showed that the latitude and the host tree species had a limited effect on the diversity and composition of fungal communities, which were largely explained by the environmental variables of each site and the substrate they colonize. The mean annual temperature and mean annual precipitation had a strong effect on root fungal communities, isothermality on needle fungal communities, mean temperature of the warmest quarter and precipitation of the driest month on shoot fungal communities, and precipitation seasonality on soil fungal communities. Fungal communities of both tree species are predicted to shift to habitats with a lower annual temperature amplitude and with increasing precipitation during the driest month, but the suitability of these habitats as compared to the present conditions is predicted to decrease in the future.

## 1. Introduction

Climate change as a combination of changing precipitation, warming, an altered pattern of extreme events, and a changing disturbance regime is predicted to significantly impact the structure of forest ecosystems, changing geographic ranges, species abundance, and interdependence [1].

In Europe, *P. sylvestris* and *P. abies* are among the most economically and environmentally important tree species [2,3]. *Pinus sylvestris* is one of the most widely distributed pine species in the world, which can be found all the way across Eurasia [4]. However, despite the ecological plasticity of *P. sylvestris*, it is predicted that changing climate will affect its climatic optimum in southern and central Europe [5]. Additionally, *P. sylvestris* is expected to be more susceptible to droughts in the western part of its current range, which will impair its growth [6,7]. All of this may indicate that as the climate changes, the pine will gradually disappear from its current habitats in the south and move to the north. Assuming an average temperature increase of 2.6–4.8 °C by 2100, it is likely that *P. sylvestris* may disappear from southern and central Europe [8], and other tree species, often geographically alien to European ecosystems, will take their place and negatively impact the environment [9]. As *P. sylvestris* is a principal tree species in large forest ecosystems, any changes in the distribution range or health status are likely to have great environmental and economic consequences [10]. *Picea abies* was extensively planted outside the limits of its natural range, where it can be particularly vulnerable to higher temperatures and droughts due to the shallow root system [11,12]. For this reason, it is expected that climate change will have severe consequences for the health and growth of *P. abies*. Indeed, weakened trees can be more easily attacked by pathogenic fungi such as *Heterobasidion annosum* s.l. ((FR.) Bref., 1889) or *Armillaria* spp., but especially by bark beetles [13]. Although in northern Europe, climate change is expected to increase the overall forest productivity due to a longer and warmer growing season, conditions for *P. abies* will become less favorable [14].

Over the years, our understanding of the role of microbial communities in the functioning of ecosystems, including microbes associated with trees, has improved significantly [15,16,17,18]. Plant-associated microbiota colonizes a majority of plant tissues and plays crucial roles supplying nutrients to plants, promoting seed germination, promoting plant growth, and protecting plants against biotic and abiotic stresses [19,20]. The rhizosphere is rich in microbial species, which are influenced by the deposition of plant mucilage and root exudates [21]. The phyllosphere, in contrast, is relatively poor in nutrients and exposed to extremes of temperature, radiation, and humidity [22]. Microbial inhabitants of the rhizosphere and phyllosphere (located near or on plant tissues) are considered to be epiphytes, while microbes found in plant tissues (endosphere), such as leaves, roots, or stems, are considered to be endophytes. Microbes in these niches can form beneficial, neutral, or harmful associations of varying intimacy with their host plants [20]. Thus, they are an integral part of many functions important for tree survival [20,21]. In addition, host-associated microbial communities may provide a basis for adaptation to new climatic conditions and increase plant tolerance to climatic stress [22,23]. Although aboveground (plant) and belowground (soil) microbial communities together regulate ecosystem processes and responses to change, they are still frequently studied separately [24]. However, the different sensitivities of plant microbial and soil microbial communities suggest that these communities may respond differently to environmental factors [24,25]. Therefore, establishing whether environmental changes drive parallel shifts above- and belowground is pivotal, predicting how ecosystems will respond to future global change [24]. In recent years, high-throughput sequencing has allowed exploring the taxonomic and functional diversity of microorganisms to a much larger extent than before [26]. However, in some areas, the available knowledge is still limited, especially on environmental and host effects, principles of community assembly, and microbe–microbe interactions [27].

Fungi constitute an essential part of the tree-associated microbiota [28]. Many fungi can be important to plant growth and nutrition, especially under challenging conditions, as they can increase tolerance to different abiotic and biotic stressors including heat, drought, salinity, heavy metal toxicity, pests, and pathogens [27,29,30,31]. Fungi can also be seen as bioindicators of tree health as many tree-associated fungi have diverse effects on the function of both individual trees and entire forest ecosystems [32,33]. Changes in the composition of fungal communities associated with trees may reflect not only changes in environmental conditions but also changes in their health condition and viability [34,35].

Environmental conditions are among the principal determinants that drive the formation and composition of fungal communities [36,37,38,39]. However, disturbances associated with climate change can significantly alter the composition and functioning of these communities [40]. These alterations may occur either in response to a change in the biology of the host or as a result of direct exposure of fungi to abiotic stressors [41]. Climate change can be expected to affect different trophic groups of fungi. For example, an increase in temperature is predicted to increase the enzymatic activity of saprotrophic fungi, leading to potentially more rapid degradation of organic matter and release of CO_2_; it may also create conditions for the spread and establishment of pathogens in new areas, resulting in increased economic losses [42,43,44,45,46]. Understanding how climate change may affect the tree microbiome is challenging due to the complexity and interconnectedness of factors driving this process.

To evaluate the potential impact of climate change, the latitudinal gradient was proposed as it may reflect the corresponding climate change due to gradual changes in temperature and precipitation along such gradient [47]. Furthermore, understanding the dependencies of microorganisms associated with trees to certain climatic regimes is the key to displaying their diversity and role in forest ecosystems on a regional and global scale [38]. For example, using a latitudinal gradient, it was shown that going towards the north there was a tendency for a reduction in diversity of both ectomycorrhizal fungi and leaf-associated fungal pathogens [48,49]. However, the diversity and specialization of fungi do not always follow a latitudinal gradient, which repeatedly highlights the complexity of the response of different taxa.

The aim of the present study was to determine the diversity and composition of fungal communities in different functional tissues and the rhizosphere soil of *P. sylvestris* and *P. abies* stands along the latitudinal gradient of these tree species distributions in Europe to model possible changes in fungal communities imposed by climate change. We hypothesized that fungal communities associated with *P. sylvestris* and *P. abies* will qualitatively (e.g., fungal species composition) and quantitatively (e.g., fungal species richness) respond to changes in latitude owing to site-specific adaptations of specific fungal species, and, therefore, will be differently affected by climate change.

## 2. Materials and Methods

### 2.1. Study Sites and Sampling

**Figure 1:**
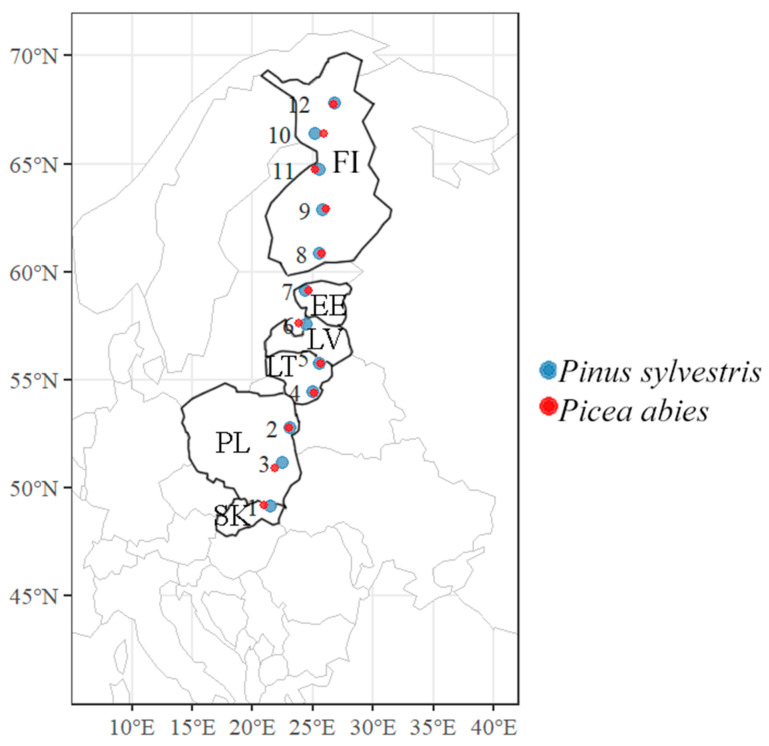
The map of central and northern Europe showing the distribution of *Pinus sylvestris* and *Picea abies* sampling sites situated along ca. 2200 km-long latitudinal gradient. Capital letters indicate the country code as follows: SK—Slovakia, PL—Poland, LT—Lithuania, LV—Latvia, EE—Estonia, and FI—Finland. Study sites are numbered as in Table 1.

Study sites were in pure 50- to 90-year-old stands of *P. sylvestris* and in pure 30- to 70-year-old stands of *P. abies* situated approximately every 200 km along the south–north gradient, i.e., between 49° and 68° latitudes and between 20° and 30° longitudes, covering countries such as Slovakia, Poland, Lithuania, Latvia, Estonia, and Finland (Figure 1 and Table 1). All study sites were situated along a 2200 km-long transect and included 12 sites in *P. sylvestris* forest stands and 12 sites in *P. abies* forest stands (Figure 1 and Table 1). Within the same sampling area, the distance between *P. sylvestris* and *P. abies* sites was not more than 5 km.

At each study site, roots, needles, shoots, and rhizosphere soil samples were collected in July and August 2019. For the collection of soil, the litter layer was removed and five random rhizosphere soil samples per site were taken separately in the vicinity (no more than 30 cm from the trunk) of five randomly selected *P. sylvestris* or *P. abies* trees using a 2 cm diameter soil core, taking at least 50 g of soil at a depth of 0–25 cm. 

Samples of fine roots were excavated in the vicinity (no more than 1 m from the trunk) of five random *P. abies* or *P. sylvestris* trees. The soil was gently removed from the roots, and each sample consisted of up to seven fine lateral roots with root tips (approximately 10 cm long). Previous year’s shoots with needles were taken in the middle part of the canopy from ten random *P. abies* or *P. sylvestris* trees using telescopic secateurs. An individual needle sample (one per tree) consisted of 25 healthy-looking needles, which were randomly collected from cut shoots using forceps. All tools were carefully cleaned between individual samples. Shoot samples were prepared by removing the remaining needles and cutting them into ca. 5 cm-long segments. Individual needle, shoot, root, and soil samples were placed in separate plastic bags and transported on ice to the laboratory, where they were kept at −20 °C before further analysis. The total number of samples for each site and tree species is shown in Table 1.

The climate data for each study site were collected from the WorldClim database (version 2.0, http://worldclim.org/version2, accessed 10 November 2021) [50]. Climate variables, which were used in the analysis, are in Section 2.4.2 and Table 2.

### 2.2. Molecular Analyses

Before DNA extraction, all samples of needles, shoots, roots, and soil were freeze-dried for 48 h using a Labconco FreeZone Benchtop Freeze Dryer (Cole-Parmer, Vernon Hills, IL, USA) and individually grounded to a fine powder using Fast prep shaker (Montigny-le-Bretonneux, France). DNA was extracted from 0.5 g (dry weight) of shoots, needles, roots, and soil samples using the CTAB protocol [53]. After DNA extraction, DNA quality, and concentration were measured using a NanoDrop™ One spectrophotometer (Thermo Scientific, Rochester, NY, USA) and adjusted to 10 ng/µL. 

The PCR amplification of the ITS2 rRNA region was performed using barcoded fungal-specific primer gITS7 [54] and barcoded universal primer ITS4 [55]. PCR was performed in 50 μL reactions containing 4 µL of DNA template. Each reaction included 1% of DreamTaq Green Polymerase (5 μ/μL) (Thermo Scientific, Waltham, MA, USA); 11% of 10× Buffer; 11% of dNTPs (10 mM); 1% of MgCl_2_ (25 mM); 2% of each primer (200 nM); and 74% of milli-Q water. Amplifications were performed using the Applied Biosystems 2720 thermal cycler (Foster City, CA, USA). The PCR was started with an initial denaturation at 95 °C for 5 min, followed by 30 cycles of 95 °C for 30 s, annealing at 56 °C for 30 s and 72 °C for 30 s, followed by a final extension step at 72 °C for 7 min. The PCR products were analyzed using gel electrophoresis on 1% agarose gels stained with Nancy-520 (Sigma-Aldrich, Stockholm, Sweden). PCR products were purified by centrifugation in 1:20 volume of 3 M sodium acetate (pH 5.2) (Applichem Gmbh, Darmstadt, Germany) and 96% ethanol mixture. An equimolar mix of all PCR products was used for high-throughput sequencing. Construction of the sequencing library and sequencing using the Pacific Biosciences RS II platform (Menlo Park, CA, USA) and one SMRT cell was performed at the SciLifeLab in Uppsala, Sweden. 

### 2.3. Bioinformatics

The sequences generated were subjected to quality control and clustering in the SCATA NGS sequencing pipeline (http://scata.mykopat.slu.se, accessed on 23 May 2021). Quality filtering of the sequences included the removal of short sequences (<200 base pairs (bp)), sequences with low read quality, dimers, and homopolymers, which collapsed to 3 bp before clustering. Sequences with a missing tag or primer were excluded. The primer and sample tags were then removed from the sequence, but information on the sequence association with the sample were stored as meta-data. The sequences were clustered into different taxa using single-linkage clustering based on 98.5% similarity. The most common genotype (real read) for each cluster was used to represent each operational taxonomic unit (OTU). For clusters containing only two sequences, a consensus sequence was produced. Fungal OTUs were taxonomically classified using PlutoF biodiversity platform (available at https://plutof.ut.ee/, accessed on 14 August 2021) and UNITE database [56]. Representative sequences of fungal nonsingletons as the Targeted Locus Study project have been deposited at DDBJ/EMBL/GenBank under accession number KHZQ00000000. FUNGuild was used to identify fungal functional groups (guilds) (FUNGuild v1.0) [36]. 

### 2.4. Statistical Analyses 

#### 2.4.1. Fungal Community Richness, Diversity, and Structure

All analyses were performed in R version 4.1.2 [57]. The comprehensiveness of the sampling was evaluated by the Good’s coverage estimator with goods function from QsRutils [36]. To estimate the relationship between the cumulative number of fungal OTUs and the number of ITS2 rRNA sequences, rarefaction analysis was carried out using iNext function from iNext [58]. To analyze factors affecting biodiversity indicators of fungal communities associated with *P. sylvestris* and *P. abies*, linear regression with a l m function was used from the stats [57]. The data were checked for multicollinearity using Variance Inflation Factor (VIF) function from regclass [59]. Variables with a high VIF (>10) were excluded from the analysis. 

A-diversity (the Shannon index, Chao 1) and β-diversity (the Bray–Curtis index) were calculated using a specnumber function to describe the abundance and richness of fungal communities using resampled OTU table. Diversity estimates were carried out using linear regression (l m function) and marginal means estimated by emmans function. Hierarchical clustering of the 30 most abundant fungal OTUs was performed using function hclust based on the Bray–Curtis distance matrix. For visualization, the heatmap function was used. A Hellinger transformation (square root of relative abundance data) was applied to the fungal community matrix to limit the influence of abundant OTUs using a function decostan from vegan package [60]. Key parameters that were influencing fungal communities associated with trees were selected using a redundancy analysis based on a function rda form vegan [60] and using a function ordistep with forward stepwise model selection using permutation tests [60], using the transformed community matrix as the response variable. Constrained Redundancy Analysis (RDA) followed by a pseudo-F test was used to assess the presence or absence of significant differences. Differences in untransformed community structure were visualized using nonmetric multidimensional scaling (NMDS) based on Bray–Curtis distance using a metaMDS function with 1000 maximum and 20 minimum number of random starts from the vegan package. The function ordistep with forward stepwise model selection using permutation tests [61].

#### 2.4.2. Climate Variables and Selection of Model for Future Predictions

For the data analysis, we use a bioclimatic variable set with a resolution of 10 min. The bioclimatic variables represented annual trends (e.g., mean annual temperature and annual precipitation), seasonality (e.g., annual range in temperature and precipitation), and extreme or limiting environmental factors (e.g., temperature of the coldest and warmest month, and precipitation of the most wet and dry quarters). In total, there were 19 different variables (Table 2). To predict the change in fungal communities over time, we used climate estimates from Coupled Model Intercomparison Project Phase 6 (CMIP6), using BCC-CSM2-MR GCM with SSP585 for the periods 2019–2040, 2019–2060, 2019–2080, and 2019–2100 with the same bioclimatic variables.

To predict the community changes, we followed Kindt (2021) [62]. The model evaluated where fungal communities would move after the change of principal bioclimatic parameters. We used the environmental.novel function, which identifies populations with future environmental conditions that are outside of the present range. Then, the probability of future conditions was calculated using pnorm with the mean and standard deviation of the present climatic conditions. If one or more variables were outside of the present range, the variable with the lowest probability was used. For the prediction and its visualization, we used the function population.shift based on RDA from AlleleShift package [62]. All visualizations of the data were based on the package ggplot2 [61].

## 3. Results

### 3.1. Sequence Quality and Fungal Diversity

**Figure 2:**
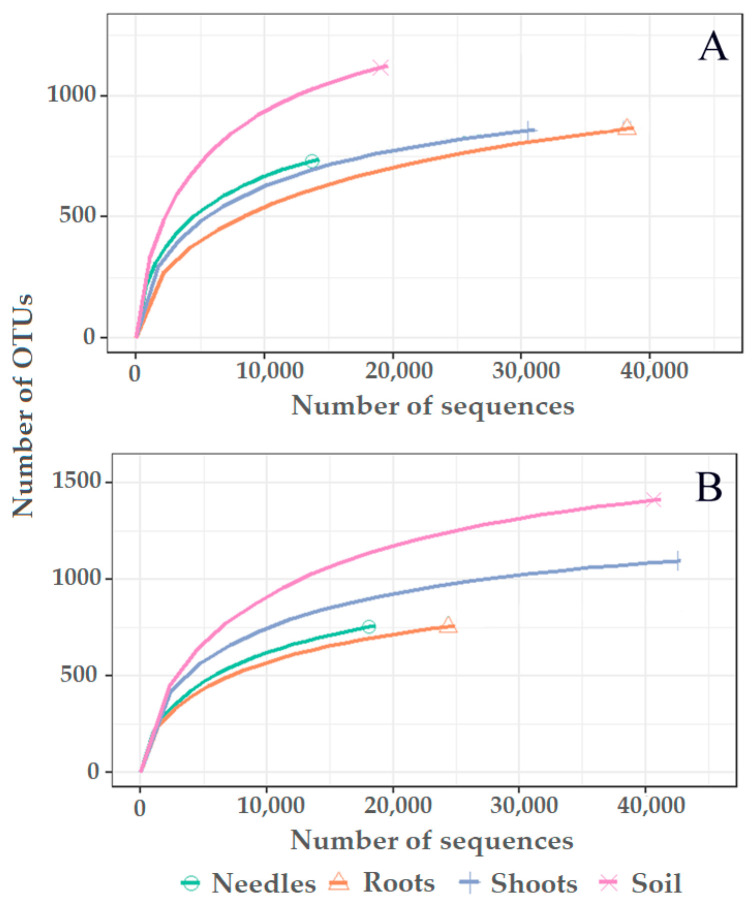
Rarefaction curves showing the relationship between the cumulative number of fungal OTUs and the number of fungal sequences from different substrates (needles, roots, shoots, and the soil) of *Pinus sylvestris* (**A**) and *Picea abies* (**B**).

High-throughput sequencing of pooled needle, shoot, root, and soil samples resulted in 349,594 reads. Filtering showed the presence of 181,342 (51.8%) high-quality reads, while 168,252 (48.2%) low-quality reads were excluded. Clustering of high-quality reads showed the presence of 3751 nonsingleton contigs at 98.5% similarity representing different OTUs, among which 3417 (91.1%) were fungal (Tables S1 and S2), which were retained, while 334 (8.9%) nonfungal OTUs were excluded. There were 2068 singletons, which were excluded. Good’s estimated coverage was >99.4%, showing that only < 0.6% of reads in individual samples appeared only once. Rarefaction analysis showed that species accumulation curves did not reach the asymptote, showing that a higher species richness could be detected with deeper sequencing (Figure 2).

Differences between the distribution of fungal OTUs associated with *P. sylvestris* (2116) and *P. abies* (2391) into different phyla was insignificant (F = 0.81, *p* = 0.54) (Tables S1 and S2). Ascomycota was the most abundant phylum, followed by Basidiomycota, Zygomycota, Chytridiomycota, and Glomeromycota, accounting for 69.0%, 24.9%, 5.7%, 0.3%, and 0.1% of sequences of *P. abies*, and 73.0%, 22.5%, 4.1%, 0.2%, and 0.2% of *P. sylvestris*, respectively. Ascomycota and Basidiomycota were significantly more abundant as compared to other phyla of both *P. sylvestris* (F = 41.54, *p* < 0.05) and *P. abies* (F = 38.14, *p* < 0.05) (Tables S1 and S2). 

**Figure 3:**
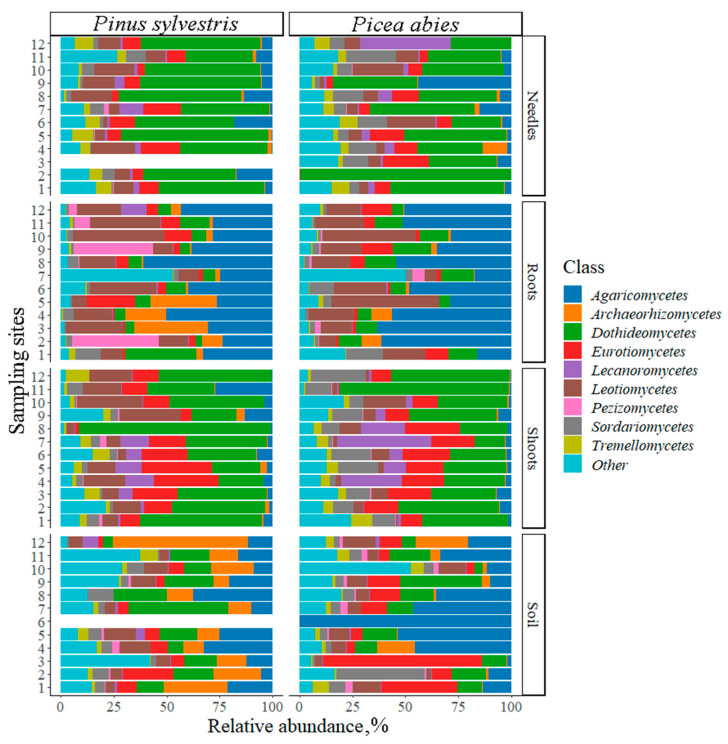
Relative abundance of fungal classes (% of fungal sequences) associated with *Pinus sylvestris* and *Picea abies*. Sampling sites: 1 (Slovakia), 2–3 (Poland), 4–5 (Lithuania), 6 (Latvia), 7 (Estonia), and 8–12 (Finland) as presented in Figure 1. Fungal classes with a relative abundance of less than 2% are marked as other.

The relative abundance of different fungal classes between *P. sylvestris* and *P. abies* did not differ significantly (F = 0.2, *p* = 0.9). The most abundant fungal classes were Dothideomycetes (27.1% of *P. sylvestris* and 33.4% of *P. abies*), Agaricomycetes (16.7% and 20.0%), Eurotiomycetes (10.6% and 12.3%), and Leotiomycetes (14.7% and 8.8%), respectively (Figure 3).

In different samples, the dominant fungal classes, respectively, for *P. sylvestris* and *P. abies* were: in needles, Dothideomycetes (47.7% and 49.3%), Eurotiomycetes (9.9% and 3.6%), and Leotiomycetes (9.3% and 510%); in shoots, Dothideomycetes (43.4% and 44.0%), Eurotiomycetes (15.1% and 12.5%), and Leotiomycetes (15.3% and 68%); in roots, Agaricomycetes (12.8% and 39.4%), Pezizomycetes (9.5% and 5.8%), and Leotiomycetes (19.3% and 18.4%); and in the soil, Agaricomycetes (20.3% and 22.7%), Dothideomycetes (21.4% and 18.9%), and Eurotiomycetes (13.3% and 22.5%) (Figure 3).

The 20 most common fungal OTUs, accounting for 35.8% of fungal sequences of *P. sylvestris* and 39.4% of *P. abies* are shown in Table 3 and Table 4, respectively. The most common fungal OTUs associated with *P. sylvestris* were *Sydowia polyspora* (5.8%) and *Acrodontium luzulae* (5.0%) (Table 3). The most common fungal OTUs associated *P. abies* were *S. polyspora* (11.5%) and *Penicillium camemberti* (4.6%) (Table 4). These most abundant fungal species were present in all sample types but in different relative abundances. For instance, in *P. sylvestris, S. polyspora* showed the highest relative abundance in shoots (15. 6%) followed by needles (3.9%), roots (0.6%), and the soil (2.4%). *A. luzulae* was abundant in the soil (11.87%) and root (7.0%) samples, but rare in needles (0.31%), and shoots (0.22%) (Table 3). In *P. abies*, *S. polyspora* was the most abundant in needles (49.7%) than in all other sample types (shoots 12.3%, roots 0.2%, or the soil 0.4%). In *P. abies*, *P. camemberti* was most abundant in the soil (12.9%) as compared with other samples (needles 0.06%, shoots 0.03%, and roots 1.93%) (Table 4). Many dominant fungal OTUs associated with *P. sylvestris* or *P. abies* at variable abundances were detected in all four substrates (Table 3 and Table 4). Some of the most common OTUs were specific to only one substrate. Unidentified sp. 5210_30 (0.95%) was only found in *P. sylvestris* root samples, while Unidentified sp. 5208_24 (2.03%), Unidentified sp. 5210_39 (0.86%), and *Piloderma lanatum* (0.81%) were only found in *P. abies* soil samples. The descriptive data for each tree species, substrate, and sampling site showing the detected number of fungal OUTs, the number of fungal sequences, and the Shannon diversity index are in Table 5.

**Figure 4:**
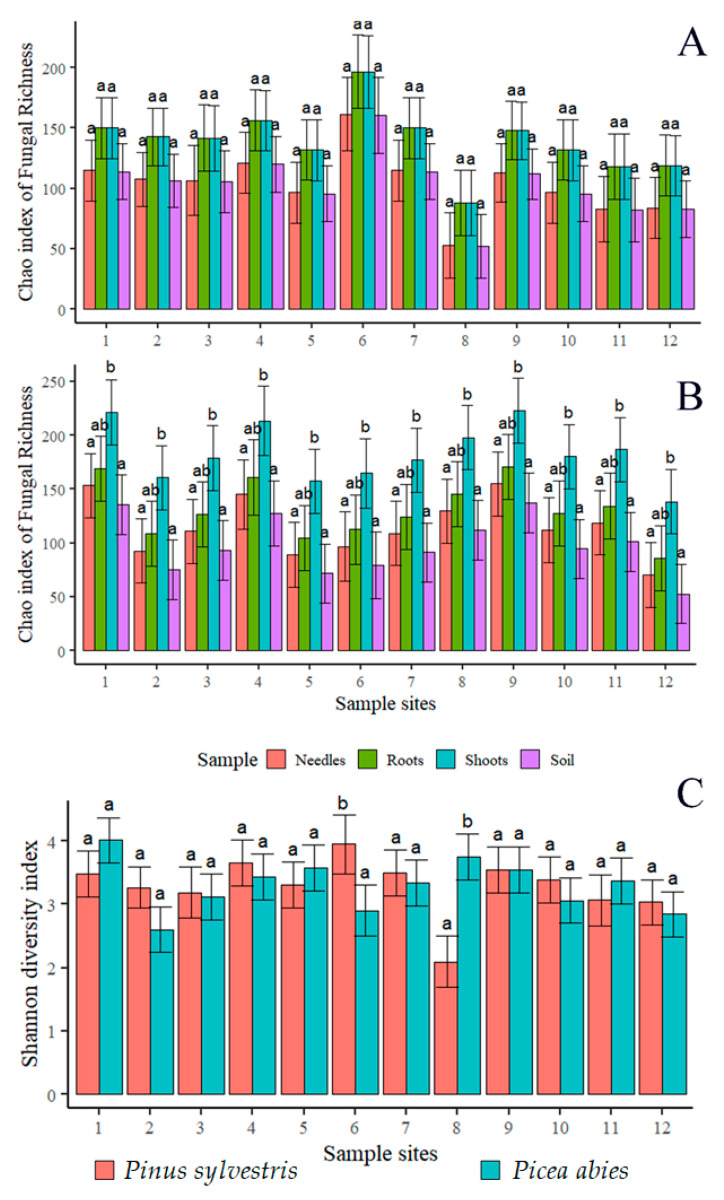
Diversity estimates: Chao1 richness index of fungal communities in different samples (needles, roots, shoots, and the soil) of *P. sylvestris* (**A**) and *P. abies* (**B**). The Shannon diversity index of fungal communities associated with *P. sylvestris* and *P. abies* (**C**). Sampling sites are as in Figure 1. Only corresponding samples (shown in the same color) were compared with each other. The same letter shows that values did not differ significantly from each other at *p* > 0.05.

The Chao1 index of fungal richness did not show significant differences among different substrates (needles, shoots, roots, and the soil) or sampling sites (*p* > 0.05), except for shoots of *P. abies*, where the species richness was significantly higher than in other substrates (Figure 4A,B). When fungal richness was compared between corresponding samples of *P. sylvestris* and *P. abies* (e.g., needles vs. needles), the difference was also insignificant. Consequently, the linear regression analysis showed that the latitude did not have a significant effect on the Chao1 index (*p* > 0.05) (Figure 4A,B). The Shannon diversity index of fungal communities associated with *P. sylvestris* and *P. abies* was in the majority of cases similar in different sampling sites (Table 5 and Figure 4C). Similarly, the linear regression analysis showed that the latitude did not have a significant effect on the Shannon diversity index (*p* > 0.05) (Table 5 and Figure 4C).

**Figure 5:**
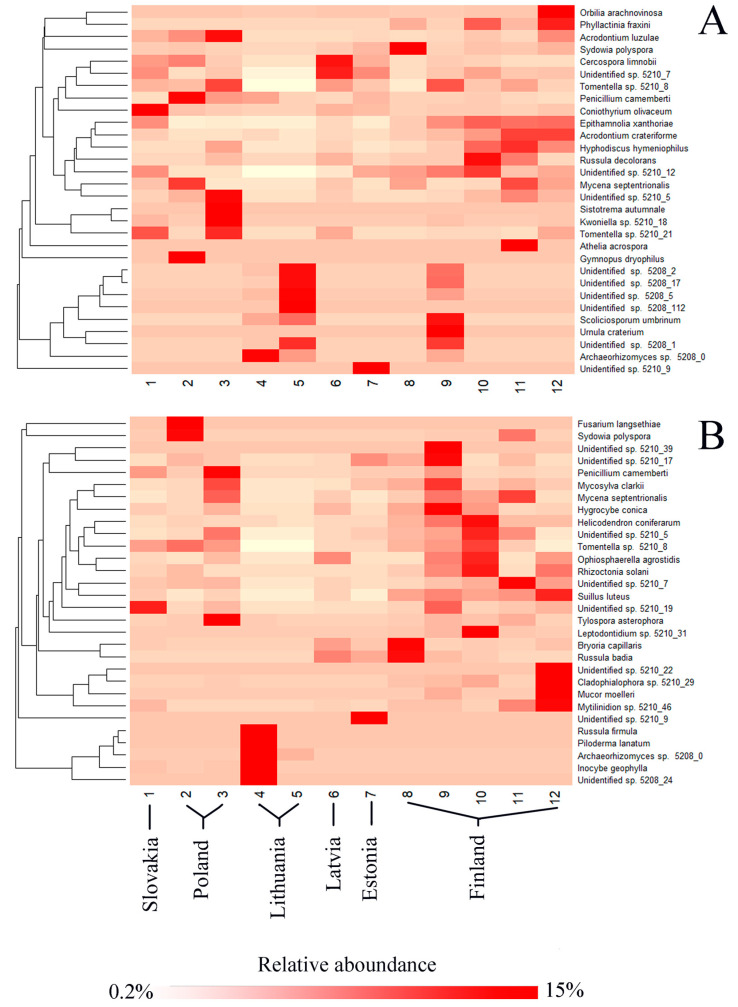
A heatmap with dendrograms showing hierarchical clustering of the 30 most abundant fungal OTUs by site (rows) and the level of species co-occurrence (columns). (**A**) *Pinus sylvestris*; (**B**) *Picea abies*.

Hierarchical clustering of the 30 most common fungal OTUs associated with each tree species showed differences in their relative abundance at different study sites (Figure 5). The analysis also revealed certain specificity of particular OTUs in different geographical regions. In *P. sylvestris*, fungal OTUs such as *A. luzulae*, *Tomentella* sp. 5210_8 or *Coniothyrium olivaceum* were more common in the southern part of the gradient, *Scoliciosporum umbrinum*, *Archaeorhizomyces* sp. 5208_0 or Unidentified sp. 5210_5 in the central part of the gradient, and *Phyllactinia fraxini*, *Acrodontium crateriforme*, or *Hyphodiscus hymeniophilus* in the northern part of the gradient (Figure 5A). In *P. abies*, fungal OTUs, such as *Fusarium langsethiae*, *P. camemberti*, or *Tylospora asterophora*, were more common in the southern part of the gradient; *Russula firmula*, *Piloderma lanatum*, or *Inocybe geophyla* in the central part of the gradient; and *Helicodendron coniferarum*, *Suillus luteus*, or *Rhizoctonia solani* in the northern part of the gradient (Figure 5B). Additionally, several OTUs, such as Unidentified sp. 5210_5, Unidentified sp. 5210_19, Unidentified sp. 5210_7, *Mycena septentrionalis*, and *Tomentella* sp. 5210_8 were common both in the southern and in the northern part of this gradient (Figure 5B).

### 3.2. Fungal Communities and Bioclimatic Factors Explaining the Community Structure

The primary analysis included temperature variables (BIO1-BIO11), precipitation variables (BIO12–BIO19), and latitude. However, latitude was excluded from further analyses as this parameter had in most cases insignificant (*p* > 0.05) effect on the Chao1 richness index and the Shannon diversity index of fungal communities (Figure 4A–C). The bioclimatic variables best explaining variance in the Chao1 richness index were the isotermality (BIO3) and mean temperature of the wettest quarter (BIO8). The primary analysis showed a strong correlation (r > 0.8) among the remaining variables representing temperature (BIO1, BIO2, BIO4-BIO11) and precipitation (BIO12-BIO14, BIO16-BIO19), which were excluded from the linear regression model. Precipitation variables (BIO12–BIO19) had an insignificant (*p* > 0.05) effect on the Shannon diversity index. The bioclimatic variables best explaining the variance in the Shannon diversity index were the isotermality (BIO3) and mean temperature of the wettest quarter (BIO8). Due to a strong correlation among other precipitation variables (BIO12-BIO14, BIO17-BIO19), these were excluded from the linear regression model. 

**Figure 6:**
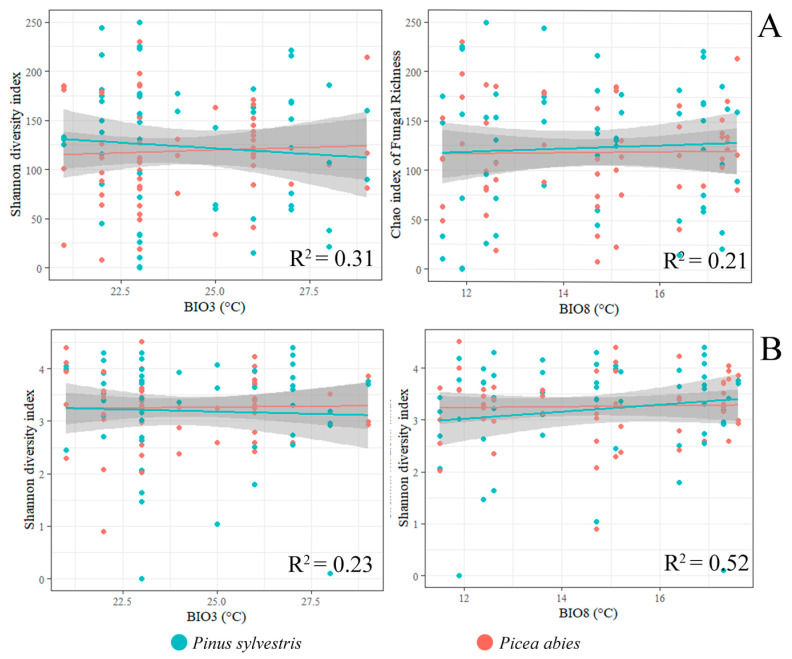
The linear regression model showing the effect of bioclimatic variables on the Chao1 richness index (**A**) and the Shannon diversity index (**B**) of fungal communities associated with *P. sylvestris* and *P. abies (p* < 0.05). The description of BIO variables is in Table 2.

For both *P. sylvestris* and *P. abies*, the linear regression model of biodiversity showed a significant (F = 7.042, R^2^ = 0.31, *p* = 0.01) effect of isothermality (BIO3) and mean temperature of the wettest quarter (BIO8) (F = 6.01, R^2^ = 0.21, *p* = 0.01) on the richness of fungal communities (Figure 6A). Both mean temperature of the wettest quarter (BIO8) (F = 2.51, R^2^ = 0.23, *p* = 0.05), and isothermality (BIO3) (F = 4.91, R^2^ = 0.52, *p* = 0.02) had a significant effect on the Shannon diversity index (Figure 6B). In general, the Chao1 richness index and the Shannon diversity index of both *P. sylvestris* and *P. abies* responded similarly to changes in isothermality (BIO3) (Figure 6A,B). By contrast, the Chao1 richness index and the Shannon diversity index of each tree species responded differently to changes in mean temperature of the wettest quarter (BIO8) (Figure 6A,B).

**Figure 7:**
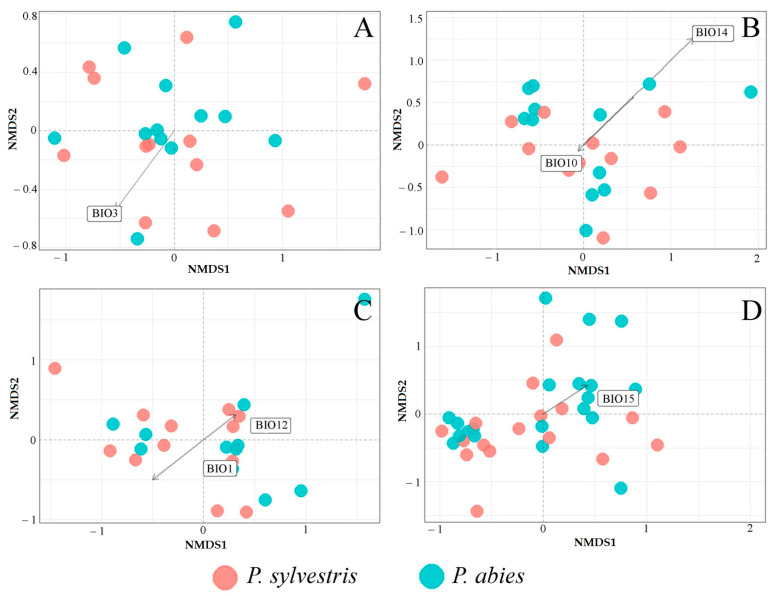
Nonmetric multidimensional scaling (NMDS) plot of fungal communities, based on the Bray–Curtis dissimilarity matrix, derived from *Pinus sylvestris* and *Picea abies* samples: (**A**) needles, (**B**) shoots, (**C**) roots, (**D**) the rhizosphere soil. Vectors show bioclimatic variables, which had a significant effect on fungal community composition (*p* < 0.05). Bioclimatic variables, which are highly correlated (R > 0.8) among each other, are not shown in the diagram. The description of bioclimatic variables is in Table 2.

MANOVA showed a significant effect (F = 4.06, *p* < 0.05) of the bioclimatic variables on fungal communities detected in different samples (needles, shoots, roots, and the soil) at *P. sylvestris* and *P. abies* study sites (Figure 7A–D). NMDS showed that isothermality (BIO3; R^2^ = 0.46, *p* = 0.04) was one of the most important factors determining the variation of fungal communities in needles of both tree species (Figure 7A). Bioclimatic variables best explaining the variance of fungal communities in shoots were the mean temperature of the warmest quarter (BIO10; R^2^ = 0.31, *p* = 0.04) and precipitation of the driest month (BIO14; R^2^ = 0.33, *p* = 0.02) (Figure 7B). However, the mean temperature of the warmest quarter (BIO10) had a more pronounced effect on *P. abies* shoot fungal communities than on these of *P. sylvestris* (Figure 7B). The bioclimatic variables best explaining the variation of fungal communities in roots were the annual mean temperature (BIO1; R^2^ = 0.21, *p* = 0.01) and annual precipitation (BIO12; R^2^ = 0.25, *p* = 0.04). Root-associated fungal communities followed both directions of decreasing annual mean temperature (BIO1) and increasing annual precipitation (BIO12) (Figure 7C). The variation of fungal communities in the rhizosphere soil was most affected by the precipitation seasonality (BIO15; R^2^ = 0.32, *p* = 0.001). Fungal communities of the rhizosphere soil of *P. abies* were largely distributed under the increasing precipitation seasonality (BIO15), while these of *P. sylvestris* were largely distributed under the decreasing precipitation seasonality (Figure 7D).

### 3.3. Projected Shifts in Fungal Communities under the CMIP6 Model

**Figure 8:**
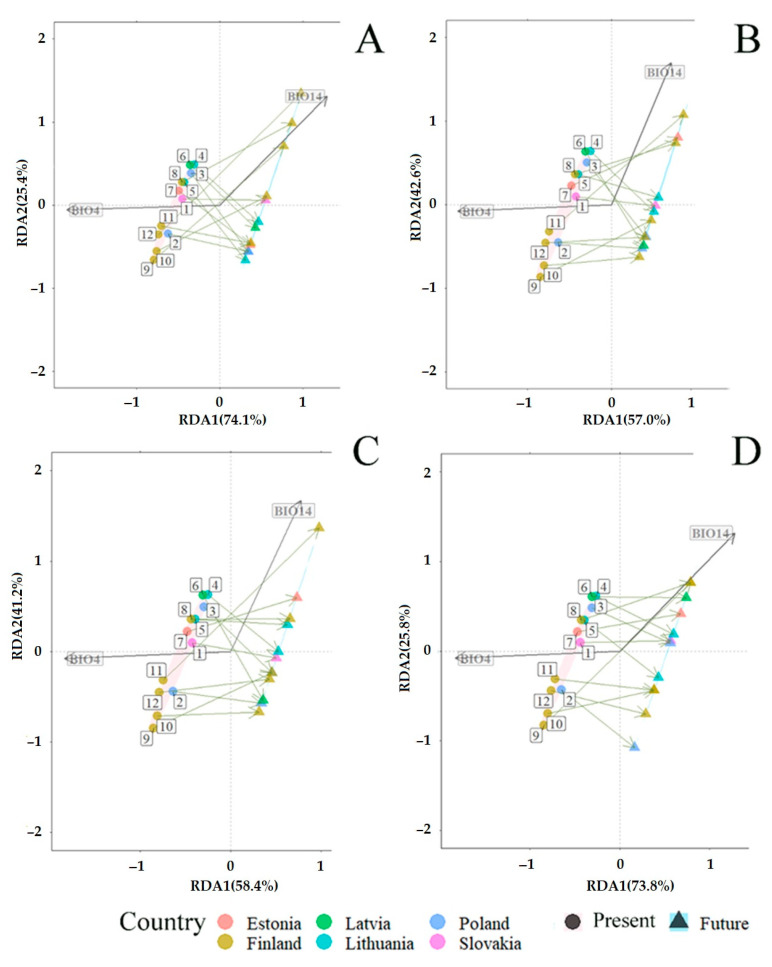
Redundancy analysis (RDA) showing the predicted changes in fungal communities associated with *Pinus sylvestris* under the CMIP6 [63] in different time periods: (**A**) 2019 vs. 2040; (**B**) 2019 vs. 2060; (**C**) 2019 vs. 2080; and (**D**) 2019 vs. 2100. Present (2019) (pink area): coordinates of sampling sites are shown by circles. The number near each circle indicates the study site: 1 (Slovakia—SK), 2–3 (Poland—PL), 4–5 (Lithuania—LT), 6 (Latvia—LV), 7 (Estonia—EE), 8–12 (Finland—FI), as presented in Figure 1. Future (blue area): expected coordinates of study sites are shown by triangles. The description of BIO parameters is in Table 2.

**Figure 9:**
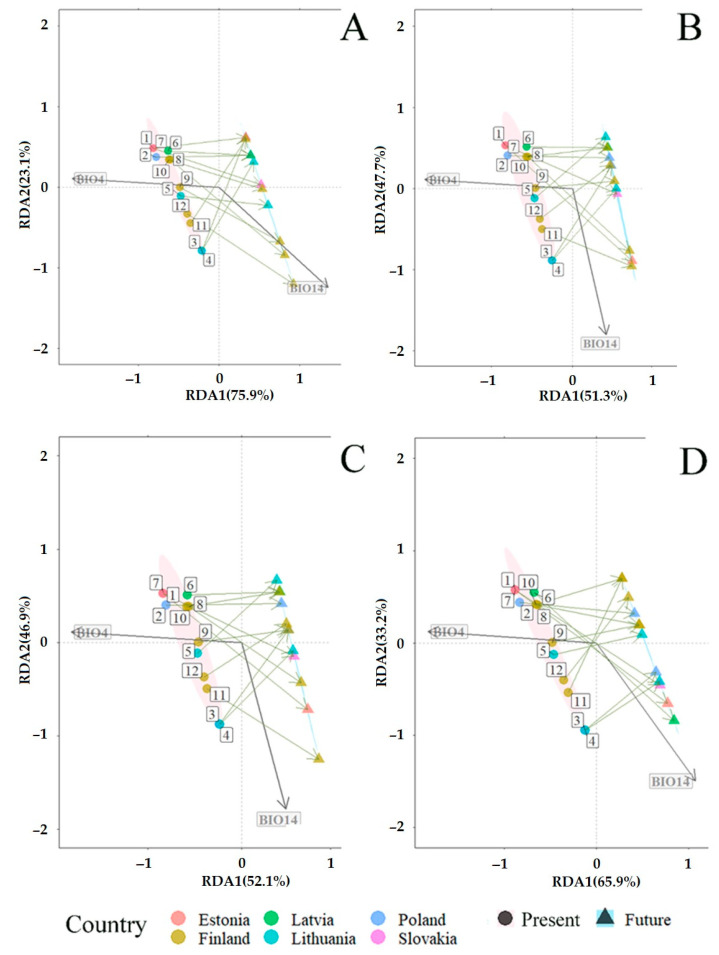
Redundancy analysis (RDA) showing the predicted changes in fungal communities associated with *Picea abies* under the CMIP6 [63] in different time periods: (**A**) 2019 vs. 2040; (**B**) 2019 vs. 2060; (**C**) 2019 vs. 2080; and (**D**) 2019 vs. 2100. Present (2019) (pink area): coordinates of study sites are shown by circles. The number near each circle indicates the study site: 1 (Slovakia—SK), 2–3 (Poland—PL), 4–5 (Lithuania—LT), 6 (Latvia—LV), 7 (Estonia—EE), 8–12 (Finland—FI), as presented in Figure 1. Future (blue area): expected coordinates of study sites are shown by triangles. The description of BIO parameters is in Table 2.

Redundancy analysis (RDA) revealed the predicted mid-term (periods 2019–2040 and 2019–2060) and long-term (periods 2019–2080 and 2019–2100) changes in fungal communities associated with *P. sylvestris* and *P. abies* (Figure 8 and Figure 9). RDA included bioclimatic variables, representing temperature (BIO1–BIO11) and precipitation (BIO12–BIO19) (Table 2), which were present at different sampling sites. Bioclimatic variables such as temperature seasonality (BIO4) and precipitation of the driest month (BIO14) best explained the variance of total fungal communities, i.e., in needles, shoots, roots, and the rhizosphere soil, they had the lowest variance of inflation factor (VIF < 10); therefore, they were used to predict changes of fungal communities associated with both *P. sylvestris* (Figure 8A–D) and *P. abies* (Figure 9A–D). In the primary analysis, the remaining variables associated with either temperature or precipitation showed a strong correlation (r > 0.8), and thus, were excluded from RDA. 

RDA showed significant (R^2^  =  0.72, *p*  <  0.001) predicted mid- and long-term changes in fungal communities associated with *P. sylvestris*. The constrained proportion variance of RDA Axis 2 explained by RDA Axis 1 was 55.9% and the unconstrained proportion unexplained variance in RDA Axis 2 was 44.1%. The RDA diagram showed that mid-term (2019–2040 and 2019–2060) and long-term (2019–2080 and 2019–2100) changes in fungal communities associated with *P. sylvestris* will be determined by the decreasing temperature seasonality (BIO4) and increasing precipitation of the driest month (BIO14) (Figure 8A–D). Therefore, when compared between individual sampling sites, fungal communities associated with *P. sylvestris* are expected to change differently. During the period of 2019–2040, fungal communities at the sampling sites 1 (SK), 8–10, and 12 (FI) are predicted to be more affected by increasing precipitation of the driest month (BIO14) than by decreasing temperature seasonality (BIO4). On the contrary, fungal communities at all other sampling sites, i.e., 2–3 (PL), 4–5 (LT), 6 (LV), 7 (EE), and 11 (FI), are predicted to be more affected by decreasing temperature seasonality (BIO4) than by increasing precipitation of the driest month (BIO14) (Figure 8A).

During the period 2019–2060, fungal communities at *P. sylvestris* study sites such as seven (EE), eight, and 11 (FI) are predicted to change depending on increasing precipitation of the driest month (BIO14), while fungal communities at other sites, i.e., 1 (SK), 2–3 (PL), 4–5 (LT), 6 (LV), 9–10, and 12 (FI), are predicted to follow the decreasing temperature seasonality (BIO4) (Figure 8B). During the period 2019–2080, fungal communities detected at 4–5 (LT), 7 (EE), 8, and 11 (FI) study sites are predicted to follow increasing vectors of BIO14, while these at study sites, such as 1 (SK), 2–3 (PL), 6 (LV), 9–10, and 12 (FI), are predicted to follow decreasing temperature seasonality (BIO4) (Figure 8C). During the period 2019–2100, fungal communities at the study sites, such as one (SK), 3three (PL), four (LT), six (LV), seven (EE), and eight (FI), are predicted to follow increasing BIO14, while these at all other study sites, i.e., 2 (PL), 5 (LT), and 9–12 (FI), are predicted to change following the decrease of BIO4 (Figure 8D). These results show that fungal communities associated with *P. sylvestris* at northern study sites during the mid-term period (2019–2040 and 2019–2060) are predicted to change depending on the increase of precipitation of the driest month (BIO14), while these during the long-term period (2019–2080 and 2019–2100) are predicted to change depending on the decrease of temperature seasonality (BIO4). Fungal communities at the southern study sites did not show a consistent trend for changes during mid- and long-term periods (Figure 8A–D). 

RDA also showed significant (R^2^  =  0.65, *p*  <  0.001) predicted mid- and long-term changes in fungal communities associated with *P. abies*. The constrained proportion variance of RDA Axis 2 explained by RDA Axis 1 was 65.8% and the unconstrained proportion unexplained variance in RDA Axis 2 was 34.1%. Mid- and long-term (2019–2040, 2019–2060, 2019–2080 and 2019–2100) changes in fungal communities associated with *P. abies* are predicted to take place similarly as in *P. sylvestris* fungal communities, i.e., under the conditions of decreasing temperature seasonality (BIO4) and increasing precipitations of the driest month (BIO14) (Figure 9A–D). However, the vector of variable BIO14 changed its direction as compared with RDA of *P. sylvestris* (Figure 8A–D vs. Figure 9A–D). This can probably be explained by the scaling resolution of the WordClim database, which was used for RDA. During the period 2019–2040, fungal communities associated with *P. abies* at the study sites 5 (LT), 8–9, and 12 (FI) are predicted to follow the increasing vector of BIO14, while these at other sites, i.e., 1 (SK), 2–3 (PL), 4 (LT), 6 (LV), 7 (EE), and 10–11 (FI), are predicted to be distributed under conditions of decreasing temperature seasonality (Figure 9B).

During both periods 2019–2060 and 2019–2080, fungal communities at the study sites, such as seven (EE), eight, and 11 (FI), are predicted to be strongly affected by increasing precipitation of the driest month (BIO14), while these at other study sites, i.e., 1 (SK), 2–3 (PL), 4–5 (LT), 6 (LV), 9–10, and 12 (FI), are predicted to be dependent on the decrease in temperature seasonality (BIO4) (Figure 9 B–C). During the period 2019–2100, fungal communities at sites, such as one (SK), three (PL), four (LT), six (LV), and seven (EE), are predicted to be strongly affected by increasing precipitation of the driest month (BIO14), while these at other study sites, i.e., 2 (PL), 5 (LT), and 8–12 (FI), are predicted to be dependent on the decrease in temperature seasonality (BIO4) (Figure 9D). These results show a more stable situation in fungal communities associated with *P. abies* during the periods of 2019–2060 and 2019–2080 (Figure 9B–C). Therefore, the key factor in the mid- and long-term changes in fungal communities along the south–north gradient of *P. abies* is predicted to be the decrease in temperature seasonality (BIO4) (Figure 9A–D). 

Results also showed that as compared to the present climatic conditions, the habitat area of fungal communities associated with both *P. sylvestris* and *P. abies* is predicted to narrow down in the future (Figure 8A–D and Figure 9A–D).

## 4. Discussion

Fungal communities associated with functional tissues and the rhizosphere soil of host trees are increasingly recognized as important factors determining forest health and sustainability in the process of climate change [36,63,64]. Furthermore, geographical gradients can serve as a useful tool providing valuable insights not only into the assembly of these fungal communities, their relationship with vegetation, climate, and soil over certain areas, but also into overall ecosystem functioning [65]. By incorporating the data on fungal communities associated with *P. sylvestris* and *P. abies* from a large geographical area and a number of bioclimatic variables, the present study provided new insights into qualitative and quantitative aspects of these fungal communities and their possible changes in the mid- and long-term perspective.

The results showed that the latitude did not have a significant effect on the diversity of fungal communities, thus, in this respect rejecting the hypothesis. In support, several studies showed that fungal diversity did not follow a general pattern of spatial distribution. For example, Wang et al. [64] showed that the Chao1 richness index and the Shannon diversity index of fungal communities in soil samples did not follow geographical coordinates; instead, these were more dependent on local site conditions. Furthermore, Tedersoo et al. [66] and Liu et al. [67] showed that fungal diversity decreased with the increase of latitude, whereas Shi et al. [68] found that fungal diversity peaked at mid-latitudes and descended towards high and low latitudes.

In the present study, a certain dependence on the latitude was revealed for several of the most-abundant fungal OTUs associated with a specific tree species (Figure 5). Consequently, hierarchical clustering showed that several OTUs of *P. sylvestris* or *P. abies* were more specifically associated with either southern, central, or northern parts of the latitudinal gradient of this study (Figure 5). Nevertheless, these results should be interpreted with caution as observed patterns may be dependent on several factors and not on the latitude alone. Several studies have also examined latitudinal clines on the diversity of fungal communities associated with a specific host species. However, the existing reports on the dependence of fungal diversity on the latitude are contradictory, including studies showing the increase [69], the decrease [28], or no change in, e.g., the foliar fungal community [70,71]. Similarly, the effect of the latitude on the fungal community composition are also variable. For example, foliar fungal communities in needles of *P. sylvestris* and *Pinus albicaulis* changed with the change of latitude [70,72], while Allen et al. [71] reported the lack of such change in the foliar fungal community of *Phragamites australis*.

A number of bioclimatic variables had a significant effect on the diversity of fungal communities, suggesting that alterations in these variables imposed by climate change is likely to have a strong impact on fungal communities associated with *P. sylvestris* and *P. abies*. Understanding the mechanisms underlying community assembly is essential for predicting compositional responses to changing environments [65]. At the same time, existing studies often emphasize the role of environmental variables as one of the main driving factors determining the abundance and composition of fungal communities [73,74]. Therefore, the integration of several bioclimatic variables representing temperature (BIO1-BIO11) and precipitation (BIO12-BIO19) (Table 2) in fungal community modeling revealed their relative importance determining fungal species richness (Figure 6A,B), diversity (Figure 6C), and fungal community composition associated with needles, shoots, roots, and the rhizosphere soil of each tree species (Figure 7A–D). Interestingly, the same bioclimatic variables often similarly explained variation in fungal communities associated with both *P. sylvestris* and *P. abies*, thereby showing a low host specificity. U’Ren et al. [37] emphasized that one of the principal components affecting the composition and functioning of fungal communities is the host plant. Nevertheless, the results of the present study showed that fungal communities associated with *P. sylvestris* and *P. abies* were similar, and only to a lower extent affected by the host plant. Therefore, the results demonstrated that the richness and diversity of fungal communities associated with both tree species responded similarly to changes in isothermality (BIO3), but a more specific response was in fungal communities of each tree species to mean temperature of the wettest quarter (BIO8) (Figure 6A,B). According to the interpretation of bioclimatic variables by O’Donnell and Ignizio [75], these results suggest that fungal richness increases when the diurnal temperature range approximates the annual temperature range, while fungal richness decreased with the excess of water during the nongrowing season and limited water supply during the growing season. Talley et al. [76] showed that there was a negative correlation between fungal richness and temperature.

The results also showed that bioclimatic variables, including temperature and precipitation, had a significant effect on the distribution variability of fungal communities associated with both *P. sylvestris* and *P. abies* (Figure 7A–D). However, there were different bioclimatic variables that explained fungal communities in different samples. For example, the mean annual temperature (BIO1) and mean annual precipitation (BIO12) had a strong effect on root fungal communities (Figure 7C), isothermality (BIO3) on needle fungal communities, mean temperature of the warmest quarter (BIO10) and precipitation of the driest month (BIO14) on shoot fungal communities and precipitation seasonality (BIO15) on soil fungal communities. Mean annual temperature and mean annual precipitation are referred to as being good predictors of plant and animal diversity at a continental scale [77]. Hawkins et al. [77] found that measures of energy, water, or water–energy balance explain spatial variation in richness better than other climatic and nonclimatic variables in 82 of 85 cases. Even when considered individually and in isolation, water/energy variables explain on average over 60% of the variation in the richness of a wide range of plant and animal groups [77]. Similar bioclimatic variables were tested by Větrovský et al. [78] showing that the mean temperature of the driest quarter, precipitation seasonality, mean temperature of the wettest quarter, precipitation of the coldest quarter, and diurnal temperature range were among the strongest predictors of individual species distributions. Fungi respond directly to rainfall levels, with more abundant, diverse, and consistent communities predominating under drought conditions, and less abundant, less diverse, and more variable communities emerging during wetter periods. The repeated recovery of fungal diversity and abundance during periodic drought events suggests that species with a wide range of environmental tolerances coexist in this community, consistent with a storage effect in soil fungi. Increased diversity during dry periods further suggests that drought stress moderates competition among fungal taxa [79]. Taken together and in agreement with the results of the present study, climate was shown to be one of the most important drivers of belowground [80,81] and aboveground microbial communities associated with the host tree [70]. 

In recent years, modeling the response of biodiversity to climate change has become an important research area [82,83,84]. For example, Alkhalifah et al. [84] have used the same bioclimatic variables as in the present study to predict changes in the distribution of *Fusarium oxysporum* causing vascular wilt disease for several crops for two time periods spanning until 2050 and 2070. The distribution of *F. oxysporum* and the suitability of its habitats was determined by bioclimatic variables such as annual mean temperature (BIO1), temperature annual range (BIO7), annual precipitation (BIO12), mean diurnal range (BIO2), and precipitation of the driest month (BIO14). The results of the present study suggest that temperature seasonality (BIO4) and precipitation of the driest month (BIO14) will be the key factors determining future changes in fungal communities associated *P. sylvestris* and *P. abies* (Figure 8 and Figure 9). The latter observations suggest that specific fungal species may require habitats with more specific climatic conditions than fungal communities overall. Our results also suggest that in a mid- and long-term perspective, fungal communities of both *P. sylvestris* and *P. abies* are likely to shift to habitats with a lower annual temperature amplitude, i.e., with temperatures becoming more similar between winter, spring, summer, and autumn seasons and with the increasing amounts of precipitation in the driest month (Figure 8 and Figure 9). These findings are consistent with general scenarios of climate change. For example, Lee et al. [85] predicted that during winter and summer periods, the change in temperature will have spatial gradients with the strongest warming in the northeast of the Baltic Sea region. The decrease in temperature seasonality is expected to be due to more pronounced winter warming than summer warming, which is likely to favor fungal communities of *P. sylvestris* in the northern study sites during the long-term period (2019–2080 and 2019–2100) (Figure 8C,D). By contrast, fungal communities associated with *P. sylvestris* at the southern study sites did not show a consistent trend for changes during mid- and long-term periods (Figure 8A–D), probably due to a relatively lower warming effect in the southern part of the latitudinal gradient. Fungal communities associated with *P. sylvestris* at northern study sites during the mid-term period (2019–2040 and 2019–2060) is predicted to change depending on increasing rainfall and especially during the drought periods, which deviates from climate change models predicting that the global hydrological cycle to become more intense [86], leading to increased precipitation in the northern Europe and decreased precipitation in the southern Europe, both in winter and in summer [85]. However, these changes in precipitation between northern and southern Europe are expected to be relatively small (e.g., Silén et al. [87]). Changes in fungal communities of *P. abies* are expected to follow a similar scenario as of *P. sylvestris* with the key predictor being temperature seasonality (Figure 9A–D). Previous studies demonstrated that fungi responded directly to rainfall levels, with a more abundant, diverse, and consistent communities predominating under drought conditions, and a less abundant, less diverse, and more variable communities emerging during wetter periods [88]. Although our results showed that changes in fungal communities will follow the trend of predicted changes in temperature and precipitation, in the predictions of global warming, the effect of high temperature can be alleviated by seasonal acclimatization, emphasizing the importance of physiological plasticity on both long- and short-term temporal scales in evaluating and forecasting vulnerability of organisms, including fungi, to climate change [89]. This may suggest that organisms, including fungi, should adapt to changing environmental conditions, which is demonstrated by our results, showing a notable reduction in the habitat area with suitable climatic conditions for fungal communities associated with both *P. sylvestris* and *P. abies* (Figure 8A–D and Figure 9A–D). 

For both *P. sylvestris* and *P. abies*, in different study sites and substrates the most abundant fungal phyla were Ascomycota and Basidiomycota, which are known to dominate among fungi colonizing soils and terrestrial plant tissues [74]. In agreement with other studies, our results demonstrated high ecological importance and wide distribution of these phyla in soils (e.g., Wei et al. [35]; Zheng et al. [65]), roots (e.g., Zhao et al. [90]), needles (e.g., Agan et al. [91]) and shoots (e.g., Sanz-Ros et al. [92]). The results also showed the predominant occurrence of fungal classes such as Dothideomycetes, Agaricomycetes, Eurotiomycetes, and Leotiomycetes (Figure 3 and Table 3 and Table 4), which are commonly reported classes from environmental samples [92,93,94]. Dothideomycetes is known to be one of the largest classes within Ascomycota, containing over 19,000 species of saprotrophs, parasites, and occasional lichen-forming species [95,96,97]. Dothideomycetes were reported from different ecosystems, ranging from hot deserts [98] to low-temperature environments in Antarctica [99,100]. Agaricomycetes was the second most-abundant fungal class, representatives of which possess several important ecological functions such as the formation of ectomycorrhizal symbiosis and wood decay in shrubs, perennial plants, and trees [101,102]. Their activity is essential for ecosystem functioning in different environments including forest soil [97,103,104] or grassland soils [105]), but also in different tissues of *P. sylvestris* and *P. abies* as was shown in the present study. Eurotiomycetes and Leotiomycetes include saprotrophic, biotrophic, lichen-forming fungi, ectomycorrhizal fungi, and endophytes [106,107], demonstrating their importance in different environments of managed and natural ecosystems.

In both *P. sylvestris* and *P. abies* samples, *S. polyspora*, a widespread saprotrophic and/or pathogenic [108] species living on conifers [109], was identified as the most common fungal species. This fungus, as one of the most common in pine and spruce tissues, was also identified during previous studies [74,110]. Although *S. polyspora* often occurs as an epiphyte or endophyte of conifers [111], some authors suggest that the fungus can become pathogenic in a colonized host under the influence of certain abiotic and biotic factors [112]. This fungus was also associated with current season needle necrosis (CSNN) on fir (*Abies* spp.) and *Pinus* sp. in the USA and Europe, developing brown bands or spots on needles, turning them reddish brown (necrotic) and even causing their shedding [113]. *S. polyspora* was also isolated from damaged needles of *Pinus yunnanensis* Franch. in Southwestern China [109], and *Pinus halepensis* Mill. in Italy [114]. Recently, it was found that *S. polyspora* can cause a rapid decline of *A. concolor* trees under abiotic stress [108]. Pan et al., [109] also indicate that *S. polyspora* has an association with bark beetles but this association appears to be not specific. The authors also suggest that the frequency of occurrence of *S. polyspora* may be related to the local environment and host species. This should be noted, especially because the fungus can change its lifestyle from endophytic to pathogenic when the climate changes.

*A. luzulae* was identified as one of the most common fungi in spruce samples, which is known as a cosmopolitan saprotrophic species of fungicolous fungi or plant decomposers [115]. The fungus was isolated from dead *Luzula sylvatica* leaves in England, rust on *Carex* sp. in Netherlands, and from *Annulohypoxylon* sp. and *Hypoxylon* sp. in Japan [115,116]. The fungus was also recorded on *M. hypericorum* rust in Iran. This was not only the first record in Iran, but also in Asia [117]. However, there is not much information about the role and significance of this species in forests or other ecosystems, therefore its role in *P. sylvestris* stands is difficult to predict. Another fungus found at a high frequency was *Penicillium camemberti,* which is a well-known fungus in the food industry, most often used in the production of Camembert and Brie cheeses, on which colonies of the fungus form a white crust [118]. Although *P. camemberti* is described as a strictly aerobic fungus that only grows on the surface of the cheese [119], it appears that it can also be found in *P. abies* soils. In a study by de Melo et al. [120] in Brazil, *P. camemberti* was isolated from soil samples collected in Cerrado State Park. The fungus was previously also isolated from soil samples collected in Söğütlük Forests of Edirne City, in Turkey [121], as well as in different *P. sylvestris* stands in Lithuania [74,122]. Although *P. camemberti* was found in soils in various forests and other natural ecosystems, its role remains largely unknown and requires further attention. 

Among the other most commonly identified fungal species, there were some species that are well-known in forests, such as *Suillus luteus* which is a widespread species in coniferous forests that forms ectomycorrhizal associations with pines including *P. sylvestris* [123,124,125] and could increase the uptake of nutrients and water and subsequently promote the growth of symbiotic plants [126]. *Mucor moelleri* was another commonly detected species, which is a common fast-growing fungus usually found in soil but can also be detected on plant surfaces. Currently, the fungus is widely studied for its antagonistic potential and as a plant growth-promoting agent [127,128,129].

## 5. Conclusions

The expected changes in the biodiversity indicators and the structure of fungal communities were not clearly traced in this study with increasing latitude. Fungal communities associated with both *P. sylvestris* and *P. abies* showed quantitative and qualitative differences based on the substrate (needles, shoots, roots, or the soil) and sampling site rather than based on the host tree species. Fungal communities in different substrates were affected by different bioclimatic variables. Under the pressure of climate change, fungal communities are predicted to shift in the future, but vectors of this shift are likely to be site-specific.

## Figures and Tables

**Table 1:** *Pinus sylvestris* and *Picea abies* sampling sites and samples collected.

Site no.	Country	Latitude	Longitude	Habitat/ Vegetation Type *	Tree Age, y	Sample Size, Units			
						Needles	Shoots	Roots	Soil
***Pinus sylvestris***									
**1**	Slovakia	49.12828	21.52911	Nb/vm	80	10	10	5	10
**2**	Poland	51.14678	22.51703	Nc/ox	70	10	10	5	10
**3**	Poland	52.77492	23.08233	Nc/ox	50	10	10	5	10
**4**	Lithuania	54.42183	24.95956	Lbl/m	65	10	10	5	10
**5**	Lithuania	56.05589	25.70758	Nbl/vm	70	10	10	5	10
**6**	Latvia	57.57453	24.43836	Nb/vm	90	10	10	5	10
**7**	Estonia	59.12831	24.42283	Nb/vm	70	10	10	5	10
**8**	Finland	60.86994	25.53239	Nb/vm	60	10	10	5	10
**9**	Finland	62.88594	25.82100	Nc/hox	70	10	10	5	10
**10**	Finland	64.71364	25.57986	Nb/vm	60	10	10	5	10
**11**	Finland	66.37572	25.21342	Na/v	80	10	10	5	10
**12**	Finland	67.80269	26.78244	Lb/m	60	10	10	5	10
**Total *P. sylvestris***						**120**	**120**	**60**	**1** **20**
***Picea abies***									
**1**	Slovakia	49.22103	21.57144	Nc/ox	30	10	10	5	10
**2**	Poland	50.93917	22.30369	Nc/hox	60	10	10	5	10
**3**	Poland	52.76811	23.07808	Lc/mox	50	10	10	5	10
**4**	Lithuania	54.42106	24.95953	Lcp/mox	60	10	10	5	10
**5**	Lithuania	56.05342	23.67956	Ncl/ox	68	10	10	5	10
**6**	Latvia	57.60172	24.40689	Lc/mox	70	10	10	5	10
**7**	Estonia	59.12831	24.42283	Nc/hox	60	10	10	5	10
**8**	Finland	60.86961	25.53197	Nc/ox	50	10	10	5	10
**9**	Finland	62.90411	25.80914	Lc/mox	60	10	10	5	10
**10**	Finland	64.71394	25.57769	Nb/vm	50	10	10	5	10
**11**	Finland	66.37719	25.27164	Lc/mox	60	10	10	5	10
**12**	Finland	67.76231	26.76128	Pc/fils	70	10	10	5	10
**Total *P. abies***						**120**	**120**	**60**	**12** **0**

* Soil topology: N—normal water availability; L—temporary water-logged soils; P—wetlands. b: poor fertility; c: moderate fertility; d: high fertility. l: light soil texture; s: heavy soils; p: binary soils [51]. Vegetation type: ox—oxalidosum, oxn—oxalido-nemoroso-piceetum, m—myrtillosum, mox—myrtillo-oxalidosum, v—vacciniosum, hox—hepatico-oxalidosum, fils—filipendulo-mixtoherbosum [52].

**Table 2:** Codes of bioclimatic variables according to WordClim database (version 2.0, http://worldclim.org/version2, accessed 10 November 2021) [50].

Code	Bioclimatic Variable
BIO1	Annual mean temperature
BIO2	Mean diurnal range (mean of monthly, maximum temperature—minimum temperature)
BIO3	Isothermality (BIO2/NIO7) (×100)
BIO4	Temperature seasonality (standard deviation ×100)
BIO5	Maximum temperature of warmest month
BIO6	Minimum temperature of coldest month
BIO7	Temperature annual range (BIO5-BIO6)
BIO8	Mean temperature of wettest quarter
BIO9	Mean temperature of driest quarter
BIO10	Mean temperature of warmest quarter
BIO11	Mean temperature of coldest quarter
BIO12	Annual precipitation
BIO13	Precipitation of wettest month
BIO14	Precipitation of driest month
BIO15	Precipitation seasonality (coefficient of variation)
BIO16	Precipitation of wettest quarter
BIO17	Precipitation of driest quarter
BIO18	Precipitation of warmest quarter
BIO19	Precipitation of coldest quarter

**Table 3:** Relative abundance of the 20 most common fungal OTUs sequenced from needle, shoot, root, and soil samples of *Pinus sylvestris*. The data from different sites are combined.

Phylum	Reference Sequence	Species Name	Similarity, %	Needles, %	Shoots, %	Roots, %	Soil, %	All, %
**Ascomycota**	MK762617	*Sydowia polyspora*	100	3.86	15.58	0.62	2.35	5.84
**Ascomycota**	KX287273	*Acrodontium luzulae*	100	0.31	0.22	7.00	11.87	5.04
**Ascomycota**	MT236531	Unidentified sp. 5210_4	99	-	0.003	7.07	0.04	2.65
**Ascomycota**	MG828280	Unidentified sp. 5210_5	83	0.08	0.09	6.41	0.15	2.46
**Ascomycota**	KP891398	Unidentified sp. 5208_1	100	0.58	6.17	-	0.11	1.93
**Basidiomycota**	MG597398	*Mycena septentrionalis*	100	0.03	0.00	5.05	0.04	1.90
**Ascomycota**	KP897305	Unidentified sp. 5210_12	100	7.00	2.99	0.08	0.12	1.88
**Ascomycota**	JX536159	*Gymnopus dryophilus*	100	-	0.01	4.72	0.01	1.77
**Zygomycota**	MT242128	Unidentified sp. 5210_2	100	0.23	0.01	4.38	0.02	1.67
**Ascomycota**	MG827987	Unidentified sp. 5210_9	99	-	-	3.39	0.41	1.35
**Ascomycota**	KY660851	*Phyllactinia fraxini*	100	0.51	3.93	0.01	0.05	1.25
**Ascomycota**	UDB0754226	Unidentified sp. 5208_17	100	0.66	3.13	-	0.09	1.04
**Ascomycota**	LR876918	Unidentified sp. 5210_7	100	3.06	0.95	0.43	0.75	1.00
**Ascomycota**	MN902367	Unidentified sp. 5208_2	100	1.46	2.56	0.003	0.20	1.00
**Ascomycota**	UDB0754118	Unidentified sp. 5210_30	91	-	-	2.56	-	0.95
**Ascomycota**	MH248043	*Archaeorhizomyces* sp. 5208_0	100	0.30	0.44	0.35	3.16	0.92
**Basidiomycota**	KT275603	Tomentella sp. 5210_8	99	1.40	0.75	0.47	1.47	0.87
**Ascomycota**	UDB028437	*Coniothyrium olivaceum*	100	0.80	0.57	1.34	0.07	0.79
**Basidiomycota**	UDB018458	*Tomentella* sp. 5210_21	100	-	0.003	2.08	0.01	0.78
**Zygomycota**	MN395040	*Kwoniella* sp. 5210_18	99	-	-	0.02	3.87	0.77
			**All**	**20.26**	**37.39**	**45.97**	**24.82**	**35.84**

**Table 4:** Relative abundance of the 20 most common fungal OTUs sequenced from needle, shoot, root, and soil samples of *Picea abies*. The data from different sites are combined.

Phylum	Reference Number	Species Name	Similarity, %	Needles, %	Shoots, %	Roots, %	Soil, %	All, %
**Ascomycota**	MK762617	*Sydowia polyspora*	100	49.66	12.26	0.22	0.38	11.48
**Ascomycota**	MT355566	*Penicillium camemberti*	100	0.06	0.03	1.93	12.86	4.56
**Zygomycota**	MT242128	Unidentified sp. 5210_2	100	-	0.08	15.86	0.24	3.18
**Ascomycota**	KU059580	*Suillus luteus*	99	0.88	6.71	0.03	0.05	2.42
**Basidiomycota**	MN902821	Unidentified sp. 5208_24	100	-	-	-	6.28	2.03
**Ascomycota**	LR876918	Unidentified sp. 5210_7	100	1.79	3.52	0.57	0.38	1.68
**Basidiomycota**	MG597398	*Mycena septentrionalis*	100	0.03	0.01	7.06	0.60	1.57
**Ascomycota**	MK390491	*Mucor moelleri*	100	0.01	3.93	0.02	0.01	1.34
**Ascomycota**	UDB035461	*Mycosylva clarkii*	99	0.03	0.00	1.88	2.62	1.22
**Ascomycota**	MG828280	Unidentified sp. 5210_5	83	0.11	0.06	5.03	0.34	1.12
**Ascomycota**	MN902647	Unidentified sp. 5210_17	96	0.01	0.00	3.09	1.46	1.07
**Ascomycota**	MH248043	*Archaeorhizomyces* sp. 5208_0	100	0.26	0.05	0.31	2.60	0.96
**Ascomycota**	MT595563	*Cladophialophora* sp. 5210_29	99	0.30	2.63	0.02	0.01	0.94
**Ascomycota**	MG679813	*Russula badia*	100	0.49	2.38	0.02	0.07	0.90
**Ascomycota**	MT236513	Unidentified sp. 5210_39	98	-	-	-	2.65	0.86
**Ascomycota**	LS450480	Unidentified sp. 5210_19	98	0.01	0.01	1.49	1.71	0.85
**Ascomycota**	ON963481	*Leptodontidium* sp. 5210_31	99	1.26	1.80	0.01	0.14	0.84
**Ascomycota**	MG827987	Unidentified sp. 5210_9	99	-	-	4.29	0.01	0.83
**Basidiomycota**	LR874260	*Piloderma lanatum*	100	-	-	-	2.52	0.81
**Basidiomycota**	KT275603	*Tomentella* sp. 5210_8	99	1.32	0.45	0.42	1.10	0.78
			**All**	**56.21**	**33.92**	**42.24**	**36.04**	**39.43**

**Table 5:** Biodiversity metrics in the study sites.

Site No.	Tree Species	Needles			Roots			Shoots			Soil		
		No. of Fungal OTUs	No. of Fungal Sequences	Shannon Diversity Index	No. of Fungal OTUs	No. of Fungal Sequences	Shannon Diversity Index	No. of Fungal OTUs	No. of Fungal Sequences	Shannon Diversity Index	No. of Fungal OTUs	No. of Fungal Sequences	Shannon Diversity Index
1	*P. abies*	177	956	4.30	63	173	3.76	235	2039	4.52	304	4079	3.73
2		21	8819	0.09	115	1529	2.96	178	2053	3.83	203	2206	3.36
3		149	872	415	185	5271	2.71	167	1144	4.01	114	4336	1.27
4		100	423	3.87	55	1205	2.56	178	2043	4.12	213	6816	2.98
5		155	1660	4.15	17	88	2.00	217	4355	4.15	349	11,519	4.31
6		169	1542	3.69	77	1032	2.90	245	3468	4.35	-	-	0.69
7		85	378	3.81	132	2416	3.23	186	1586	3.77	192	1743	3.66
8		197	1231	3.60	120	1913	2.40	236	3531	4.07	157	1752	3.22
9		91	873	3.05	160	2525	2.67	264	4446	3.30	291	5864	3.80
10		166	1089	2.33	102	2019	3.44	267	5526	3.90	104	685	4.47
11		78	257	1.89	162	2657	4.03	140	6810	3.79	245	1460	3.86
12		12	16	3.67	185	3528	3.44	157	5548	4.65	62	193	3.69
Total *P. abies*		752	18,116	3.38	753	24,356	4.42	1094	42,549	4.97	1408	40,653	5.07
1	*P. sylvestris*	161	1812	3.80	149	4211	3.20	119	651	3.73	208	1426	3.61
2		92	833	3.56	175	5087	2.70	36	63	3.30	279	2443	3.85
3		113	800	3.60	122	5764	2.40	184	1833	4.07	181	2276	3.21
4		92	775	3.56	45	1050	2.57	176	5462	3.51	288	4534	461
5		131	2187	3.08	25	258	2.08	196	4829	3.72	215	2734	4.42
6		208	2695	3.64	137	1472	3.57	250	1689	4.60	-	-	-
7		145	673	4.12	139	3971	2.84	183	1664	3.84	181	1191	3.54
8		70	554	4.31	121	2654	3.74	103	4336	4.52	171	1610	3.98
9		136	1329	4.15	191	2877	2.69	107	850	4.02	84	198	4.05
10		88	545	0.09	153	2538	2.91	201	2776	3.81	120	738	3.36
11		95	520	2.35	194	5331	1.01	110	2451	3.01	21	120	3.71
12		117	944	2.21	160	3012	4.13	117	3863	4.27	98	2806	4.49
Total *P. sylvestris*		731	13,667	4.94	863	38,225	4.52	855	30,467	4.63	1142	20,078	5.29

## Data Availability

The data is available upon request from the corresponding author.

## Supplementary Materials

The following supporting information can be downloaded at: https://www.mdpi.com/article/10.3390/jof9080829/s1, Table S1: Relative abundance of fungal OTUs sequenced from needle, shoot, root and soil samples of *Pinus sylvestris*.; Table S2: Relative abundance of fungal OTUs sequenced from needle, shoot, root and soil samples of *Picea abies*.
